# Enhancing Mixing and Thermal Management of Recycled Carbon Composite Systems by Torsion-Induced Phase-to-Phase Thermal and Molecular Mobility

**DOI:** 10.3390/polym12040771

**Published:** 2020-04-01

**Authors:** Ranran Jian, Zhonghe Shi, Haichao Liu, Weimin Yang, Mohini Sain

**Affiliations:** 1 College of Electromechanical Engineering, Qingdao University of Science and Technology, Qingdao 266061, Shandong Province, China; 2 Centre for Biocomposites and Biomaterial Processing, Department of Mechanical and Industrial Engineering, University of Toronto, Toronto, ON M5S 3B3, Canada; 3 College of Mechanical and Electrical Engineering, Beijing University of Chemical Technology, Beijing 100029, China

**Keywords:** field synergy, torsional flow, heat and mass transfer, recycled carbon composites, mixing and thermal management

## Abstract

A novel torsion screw has been proposed to resolve the inadequate control of mass transfer and the thermal management of two component polymer blends and their carbon fiber composites. The novel torsional screw distinctly introduced radial flow in the torsion screw channel, which is a significant improvement over the flow pattern developed by the conventional screw. The heat transfer and mixing behavior of melt mixtures are enhanced by adapting screws with torsion elements compared with the traditional screw elements. Heat transfer efficacy in the polypropylene–polystyrene bi-phasic extrusion process improved with the increase in torsion element numbers. An increased number of newly designed torsional elements also improved the dispersion of minor phase in bi-phase polypropylene–polystyrene composition and their carbon fiber composites. The unique flow pattern induced by the torsion elements shows a synergistic effect on the melt-phase mass flow and the thermal flow field facilitating phase-to-phase thermal and molecular mobility and enhanced fiber orientation, crystallinity and mechanical properties of composite made from recycled carbon fiber/polypropylene. Microtomographs of recycled carbon fiber demonstrated the extraordinary ability of a torsion screw element to orient carbon fiber in both axial and radial directions.

## 1. Introduction

Nowadays, extruder and injection molding machines are widely used for manufacturing polymer composite products. The latter cannot be separated from the fundamental principles of a screw plasticizing system, i.e., the control of plasticization under viscous flow conditions. The precise control of viscous flow dynamics from bulk to the surface combined with their coupling effect of a more uniform temperature distribution leads to systems that are more consistent in their composition, morphology and functionalities. The mixing and thermal management of polymers melts in the screw system to become the key to determining the quality of the composite products [1,2]. The effects of barrel configurations and screw designs on heat and mass transfer have been investigated for a long time and have proved to be unquestionably important attributes for determining thermal efficacy and mixing effectiveness in extrusion and injection molding processes [3,4,5,6,7,8].

Due to the high viscous dissipation of polymer melts and their low thermal conductivity, the temperature distribution in the highly viscous polymer phase can be broad [9], especially for the radial temperature differences between the barrel and the screw root. The later essentially results in unwanted heat loss and poor melt quality, and thermosensitive polymers such as biopolymers may even be degraded. Many researchers have studied several aspects of the heat transfer phenomenon of fluid by investigating viscosity changes as a function of temperature and shear rate. For example, Wei et al. [10] discussed the heat transfer behavior of polymer melt considering viscous dissipation by finite element solutions. Zhang et al. [11] also analyzed the heat transfer performance of polymer with viscous dissipation by an element-free Galerkin (EFG) method, and presented the local Nusselt number with different inlet temperatures. Both of their works infer that the viscous behavior of polymers had an important impact on heat transfer. Kaushik et al. [12] revealed that the interaction of polymer rheology and squeezing dynamics alter the Nusselt number and heat transfer characteristics. Karkri et al. [9] presented the Nusselt number in the steady laminar convective heat transfer of a polymer through an extrusion die. Their results show that the Nusselt number improves with an increasing flow rate. Estrada et al. [13] evaluated the energy efficiency and melt temperature with different plasticization processes and screw geometries, allowing a better understanding of the effect of plasticization on energy loss.

Moreover, the heat transfer is also governed by the flow patterns and the flow conditions, i.e., mass transfer and mixing [14]. In general, mixing contributes to enhanced mass transfer, and further improves heat transfer and distributes physical attributes of individual components more uniformly including temperature and viscosity. Monchatre et al. [15] investigated the temperature and frictional heat development (viscous dissipation) in a reciprocating single-screw extruder. Results indicated that a high Nusselt number is probably related to the disturbing flow (induced by the pin elements) and the pulsating flow (induced by the axial movement of the screw) to facilitate distributive mixing and active convection heat transfer. In addition, Teixeira et al. [16] developed a global plasticization modeling software to simulate the flow situations with heat transfer for polymer extrusion by the effect of operating conditions and screw geometries. In yet another work, Dhanasekharan et al. [17] also carried out a numerical simulation approach to simulate the mixing and heat transfer. The simulation results help to make a parametric design of screw configurations. Wang et al. [14] investigated the influence of four typical screws on mixing and heat transfer behavior of polymer melt by the finite element method (FEM). Kuzyaev [18] developed a mathematical model for the optimization of mass and heat transfer processes in the working channel of extruders, considering the changes in the geometrical and technological characteristics. Spina et al. [19] simulated the crystallization of isotactic polypropylene (PP) with different shear regimes and results indicated that flow had an important influence on the crystallization during polymer melting and solidification of PP. In one of our earlier works, we proposed a novel torsional screw geometry with twisted grooves, namely a torsion element (TOE), by adapting the field synergy principle to induce torsional flow in the working channel of the screw plasticization unit. The proposed design has been simulated and proved to have excellent heat transfer and mixing properties compared to a standard (STD) screw geometry [20,21]. Results indicated that the presence of the torsional flow in the working channel of TOEs induced strong distributive mixing and favored thermal exchange to obtain a high Nusselt number, which improved the synergy and interaction between velocity and temperature fields.

In this study, we determined the effect of the geometrical feature of TOEs on the heat transfer efficiency and mixing performances in the extrusion process and compared them with conventional screws by an extrusion experiment. We also examined the effect of TOE arrangement on the fundamental attributes characterizing the screws, namely, mass and heat transfer. Then the high strength reclaimed carbon fiber-based composites were prepared. Furthermore, a qualitative model was proposed for the synergy between the velocity and the thermal flow fields in a TOE channel. As far as we know, this is the first time that the heat transfer and thermal management in a viscous polymer has been investigated from the perspective of field synergy.

## 2. Experimental

### 2.1. Materials

Polypropylene (PP, T30S) used for the melt extrusion study was supplied by Sinopec Zhenhai Refining and Chemical Company (ZRCC, Ningbo, Zhengjiang, China) and used as the dominant matrix for the heat transfer and mixing experiment. High impact polystyrene (HIPS, PH-88SF) was obtained from Zhenjiang Chi Mei Chemical Co., Ltd. (Zhenjiang, Jiangsu, China) and used as polymer filler for the mixing experiment for preparation of the PP/HIPS bi-phase composite blends (PP:HIPS = 100:10, % wt/wt). Reclaimed carbon fibers (RCFs) were obtained from Ford Motor Company (Windsor, Ontario, Canada) and used as the reinforced filler for preparation of the fiber reinforced polypropylene composites (PP:RCF = 100:5, % wt/wt). The average diameter of RCFs was about 5~8 μm. Table 1 presents a summary of the physical parameters for polymers and fiber.

### 2.2. Test Stand and Torsion Element

**Figure 1:**
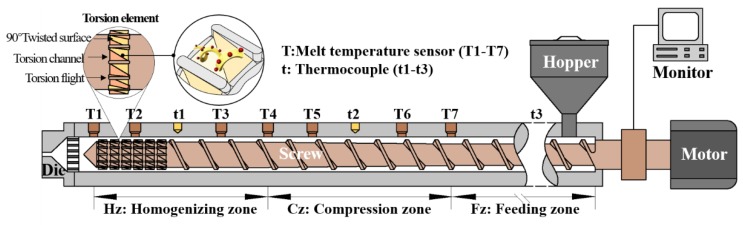
Test extruder (28D) employed in the experiments.

The extrusion process for plasticized PP and HIPS was studied by applying a filament extruder with an aspect ratio of 28:1 (Figure 1). The extruder had a modular single screw in the barrel. Seven melt temperature sensors were employed to measure the temperature of polymer melt inside of the barrel, as shown in Figure 1. In order to obtain a reliable measurement of the melt temperature and to avoid a false display of the barrel wall temperature as a melt temperature, the temperature sensors were inserted through a hole in the barrel in such a way that their ends were immersed in the melt halfway between the barrel and the screw to record the melt temperature more accurately. There are also three thermocouples in the middle of each zone of the extruder barrel to measure and control the extruder temperature. All the experiments were performed under a constant processing temperature. During the extrusion process, flow rate, melt temperature, motor current and other related variables were monitored and recorded on a computer monitor.

**Figure 2:**
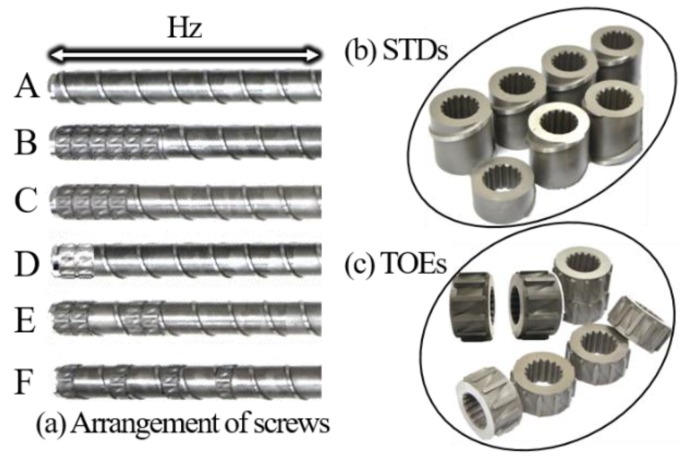
Modular screws employed in the experiments: (**a**) Arrangement of screw elements; (**b**) Standard elements (STDs); (**c**) Torsion elements (TOEs).

The geometric structures of the proposed torsion element (TOE) can be found in Figure 1 and Figure 2. The TOE is evenly separated into several torsion channels by torsion flights, and between every two adjacent torsion flights, there are two surfaces that are twisted by 90° along the axial direction. When a polymer flows over the torsion channel, it is expected to undergo a torsional rotation (tumbling) under the forces generated from viscous friction with barrel wall and with the steering between two 90° twisted surfaces. As a result, spiral-shaped flow may occur in the torsion channel.

Owing to the modular nature of the screw used in the experiments, six screw inserts with different arrangements of TOEs in the homogenizing zone (Hz) could be mounted, among which screw A (Figure 2a) was a traditional one for comparison purposes and screws B-F were torsional ones (Figure 2a). These modular screws equipped with two insert element types: STDs (Figure 2b) and TOEs (Figure 2c). Among these six screws, each of the torsional screws C, E and F had four TOEs, which were located at different axial positions. The TOEs were partially (screw E) or totally (screw F) separated by one pitch length of the STD element. The torsional screws B and D had six and two TOEs, respectively, all of which were located side by side and close to the end of the extruder head. Table 2 presents a summary of the geometric parameters for both screw elements.

### 2.3. Characterization

#### 2.3.1. Size Measurement of Dispersed Phase in Extrudates

The size of the dispersed HIPS in PP/HIPS extrudates was examined by a scanning electron microscope (Hitachi S4700) images. The average particle size (*D_n_*) was calculated using the formula (1) [22,23].

(1) $$D_{n}=\frac{\sum^{\;}N_{i}D_{i}}{\sum^{\;}N_{i}}$$

where *D_i_* and *N_i_* are the diameter and number of the particle *i*, respectively.

Coefficient of variation was applied to evaluate the variability in particle size and content of dispersed phase, which reflects the dispersion degree of parameters. It can be calculated using formula (2) [24].

(2) $$C_{v}=\frac{\sigma }{\mu }$$

where *C_v_* is the coefficient of variation, *σ* is the standard deviation (SD), and *μ* is the average value.

#### 2.3.2. Heat Transfer Property

A heat transfer coefficient was employed to measure the total heat transfer inside the extruder in this study. It can be calculated by Newton’s law of cooling, as shown in Formula (3).

(3) $$Q=\alpha A\left(T-T_{w}\right)$$

where *Q*, *A*, *T*, *T_w_* and *α* represent the heat content, the heat transfer area, the temperature of polymer melt, the temperature of the barrel wall and the heat transfer coefficient, respectively.

## 3. Results and Discussion

### 3.1. Mass Transfer and Mixing Characteristics

**Figure 3:**
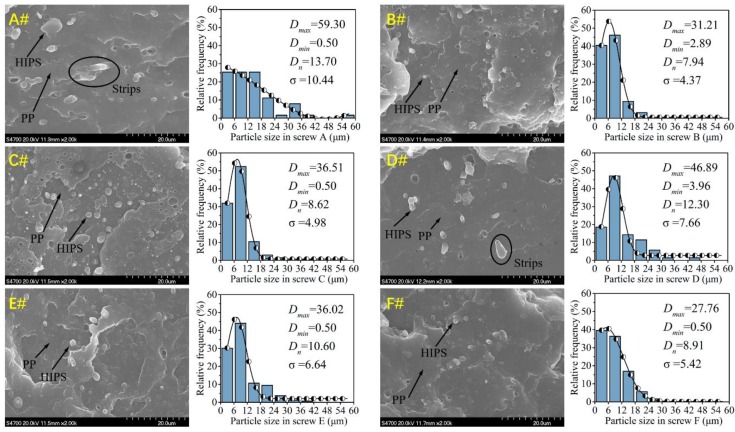
Particle size distribution of dispersed phase for various screws.

Polymer particle size distribution (HIPS dispersed in PP matrix at the extrusion filament samples) for six sets of screws can be found in Figure 3. The scanning electron microscope (SEM) images clearly show that the particle size of HIPS in screws B and F is finer and smaller than in the others. Furthermore, HIPS particles agglomerate with long strip shapes that are also observed in screws A and D, which indicates poor dispersive mixing. To better quantify the particle size of the dispersed phase, the average particle size (*D_n_*), the maximum value (*D_max_*), minimum value (*D_min_*) and standard deviation (SD) of HIPS particles were calculated from each SEM image and summarized in Figure 3. Results indicated that screws A and B have the highest and lowest *D_n_* and SD, respectively, among those six screws; screws A and F have the highest and lowest *D_max_*, respectively, among the six screws. Therefore, screw B, with six TOEs, shows the best mass transfer and mixing performances among all the screws. Besides, the particle size distribution of HIPS phase fits Gaussian distribution.

**Figure 4:**
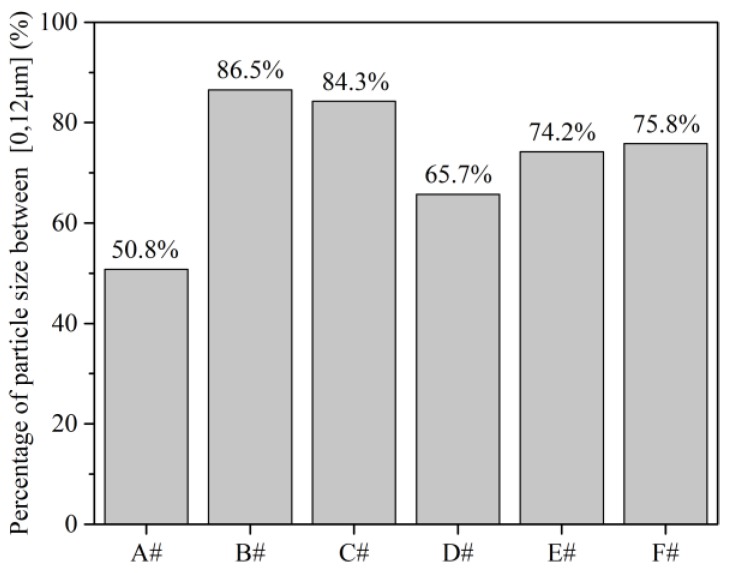
Percentage of small-size high impact polystyrene (HIPS) particles for various screws.

Figure 4 shows the percentage of HIPS particle size between 0 to12 μm for six sets of screws. It can be seen that screws A and B have the smallest and largest percentages of small-size HIPS particles, respectively, among the six screws. The percentage of small-size HIPS particles in screw A is only 50%, which is much less than that of screws B, C and F (higher than 75%). The results further indicated that the conventional screw A shows the worst dispersive mixing compared to the torsional screws.

**Figure 5:**
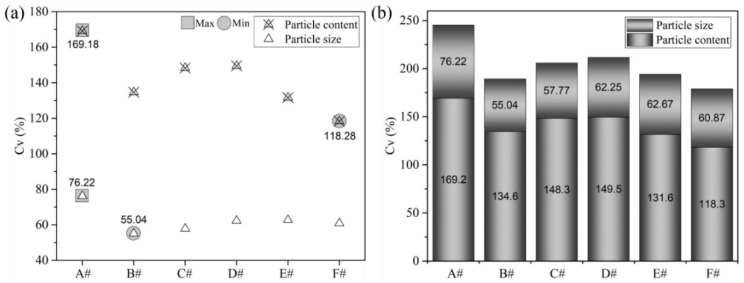
Coefficient of variation (*C_v_*) of particle size and content for various screws: (**a**) Graphs of particle size and content; (**b**) Weighted data of HIPS particle size and content.

Figure 5 shows the *C_v_* values of HIPS particle size and particle content in the PP/HIPS blend phase for six sets of screws. More specifically, Figure 5a shows the graphs of particle size and content, respectively, and Figure 5b shows the weighted data of HIPS particle size and content. For HIPS particle size, as shown in Figure 5a, we can infer that the *C_v_* of particle size decreases with an increase in the TOE number as well as with a more dispersed arrangement of the TOEs (screw F). Among these six screws, screws A and B have the largest and smallest *C_v_* of particle size, respectively. It can be inferred that the particle size fluctuation of HIPS obtained in screw B is smaller than that of others. For HIPS particle content (Figure 5a), the *C_v_* of particle content also decreases with the increase in the TOE number. The same trend is observed in a screw where TOEs are placed apart from each other, for example, screw F. Screws A and F have the largest and smallest *C_v_* of particle content, respectively. This observation is in conformity with the additive values of particle size and content (Figure 5b). Therefore, screw B and screw F show good mixing and mass transfer performance compared to other screw configurations.

### 3.2. Heat Transfer Property

**Figure 6:**
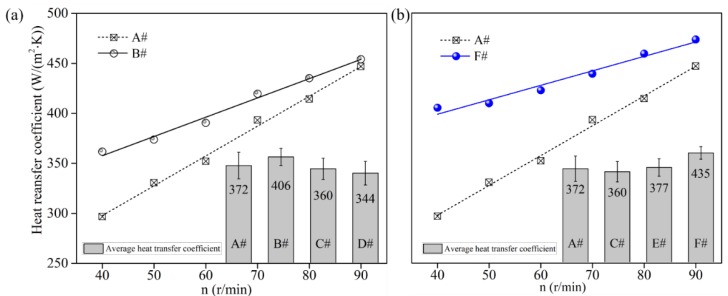
Convective heat transfer coefficient (*α*) for various screws: (**a**) Variable TOE numbers; (**b**) Dispersed arrangement of TOEs.

Heat transfer coefficient (*α*) of the PP plasticization system for six sets of screws is illustrated in Figure 6. From Figure 6a,b, we can see that the average heat transfer coefficient improves with TOE numbers (B# > C# > D#) and the dispersed arrangement of TOEs in the screw (F# > E# > C#), where the heat transfer coefficient of screw A is smaller than that of screw F, B and E. On the other hand, the heat transfer coefficient of screw A is larger than that of screw C and D. Therefore, it can be concluded that if the numbers of TOE are not high enough or the arrangement of TOEs is not sufficiently apart from each other, the heat transfer performance will hardly be improved. Furthermore, screw F has the highest heat transfer coefficient and screw B has the second highest heat transfer coefficient among all six screws. Results indicate that the arrangement of TOEs in screws has a great influence on heat transfer. The TOEs distributed with more gaps between them, i.e., more dispersed positioning of TOEs, allows a more efficient heat transfer compared to the configurations where all TOEs are placed side by side. From Figure 6, we can also find that the heat transfer coefficient of all screw plasticization systems increases with screw speed that increases the Reynolds number (Equation (4)). In addition, the heat transfer coefficients of screws B and F are higher than that of screw A at all screw speeds. Furthermore, the difference value of heat transfer coefficient between screws A and B becomes smaller and smaller with the increase in screw speed, as it does for screws A and F. A possible explanation for this deviation could be that the influence of the Reynolds number on heat transfer becomes more and more important with the increase of screw speed. As a result, we can state that the torsional-spiral flow induced by TOEs has a positive influence on convective heat transfer performance.

**Figure 7:**
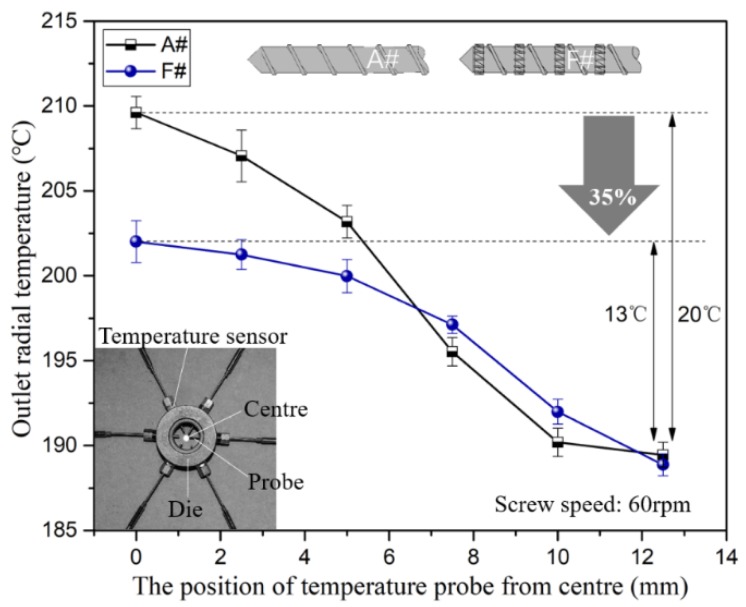
Outlet radial temperature distribution in the extrusion die.

In order to confirm the influence of TOE-configured screws on melt temperature uniformity, six of the melt temperature sensors were employed and inserted through a hole in the die with their ends immersing in different radial positions in the melt. Using screw F as an example, the outlet radial temperature distributions in the extrusion die of screw A and screw F are illustrated in Figure 7. It can be found that the melt temperature in the center of the die is higher than it is near the barrel wall. This is because of the viscous dissipation of polymers, which means that a good heat transfer is needed to facilitate the effective transfer of excess local heat out of the bulk of the polymer melt. Moreover, the radial temperature difference of screw F is 13 °C, whereas the radial temperature difference of screw A is 20 °C, which is 35% higher than that of screw F. Results indicated that the radial temperature difference of melt decreases in the TOE-configured screw owing to its good heat and mass transfer performance.

### 3.3. Fluid Flow Characteristics and Heat Transfer Mechanism

**Figure 8:**
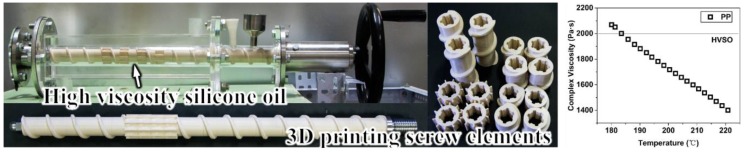
Visualization test stand: general view of extruder with visualization feature used in the experiments.

**Figure 9:**
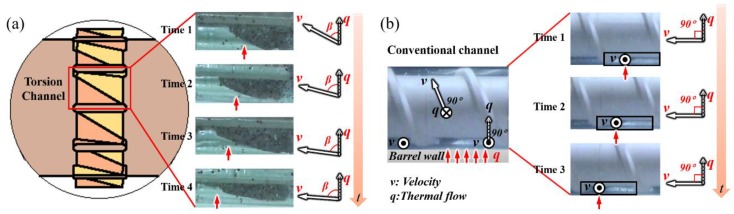
Flow patterns of fluids in the TOEs and STDs channels at different times: (**a**) TOE channel; (**b**) STD channel.

**Figure 10:**
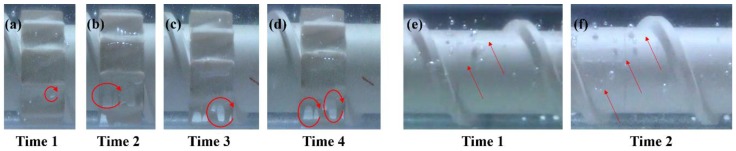
Flow patterns of bubbles in the TOEs and STDs channels at different time: (**a**) Time 1 in TOE channel; (**b**) Time 2 in TOE channel; (**c**) Time 3 in TOE channel; (**d**) Time 4 in TOE channel; (**e**) Time 1 in STD channel; (**f**) Time 2 in STD channel.

In order to confirm the improved mass transfer effect of TOE-configured screws, we simulated the flow states in both TOE and STD screws using high viscosity silicone oil (HVSO) by carrying out a test run with visualization extrusion, as shown in Figure 8. The initial kinematic viscosity of the silicone oil was 2 m^2^/s (nearly 2000 Pa·s) at room temperature, which matches the viscosity of the PP melt at the extrusion conditions (Figure 8). The screw elements were manufactured using a 3D printer. Figure 9 shows the flow patterns of fluids in TOEs and STDs channels at different time intervals. We can see that the HVSO fluid yielded ductile deformation (Figure 9a) in one TOE channel, and the velocity direction changed with time, i.e., the included angle between velocity and thermal flow fields became smaller and smaller (Equation (4)), which was not achieved in the STDs channel, as shown in Figure 9b. Figure 10 shows the bubble shape air pockets and flow patterns in the TOEs as well as in the STDs channels at different time intervals. From Figure 10a–d, it can be seen that the bubbles gradually grow, which indicates that a sharp twisting resistance mounts in the TOEs channel. However, the bubbles in the STDs channel move forward steadily along the screw flights without large deformation as shown in Figure 10e–f. All these results indicated that the torsional-spiral flow occurs in the working channel of TOE.

**Figure 11:**
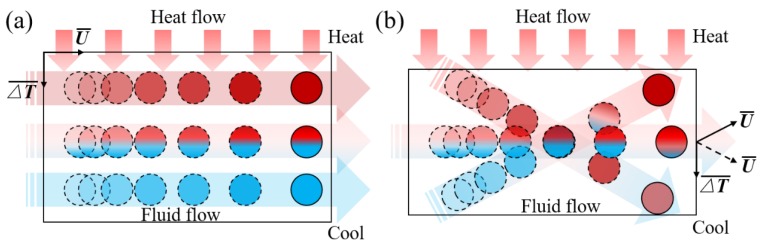
Synergy model between velocity and thermal flow fields in STD and TOE working channel: (**a**) STD channel; (**b**) TOE channel.

Based on the flow characteristics, a qualitative model for the synergy between velocity and thermal flow fields is proposed, as illustrated in Figure 11. The upper boundary is the barrel wall, which receives thermal energy from the heater, and the bottom boundary is the screw wall, whose surface temperature is lower than that of the barrel wall. From Figure 11a, it can be postulated that the fluid in the STD channel flows forward as a whole rectangle without radial position change, and the heat is transferred through polymer melt layer by layer. Owing to the poor thermal conductivity of the polymers, the fluid close to the screw wall is still cooler than the fluid near the extruder barrel surface. From Figure 11b, it can be seen that the mass transfer occurs in the radial direction due to the twisted groove in the TOE channel, i.e., the hot fluid close to the barrel wall has a chance to flow in a tangential direction, and the cold fluid close to the screw wall can move in an upward direction, thereby, ensuing a better energy transfer and heat exchange. Moreover, the included angle of the flow field and the thermal field will no longer be dominantly perpendicular, i.e., the synergy of velocity and temperature gradient will be enhanced. Because of the previously mentioned synergy, heat transfer will be enhanced in line with the field synergy principle, Equation (4) [25,26].

(4) $$RePr\int_{0}^{1}\left(\left|\bar{U}\right|\cdot \bar{\left|\nabla T\right|}\cdot cos\beta \right)d\bar{y}=Nu\;(0{}^{\circ}<\beta <90{}^{\circ})$$

where $\bar{U}$, *Re*, $\bar{\nabla T}$, *Nu*, *Pr* and *β* represent the velocity vector, the Reynolds number, the temperature gradient vector, the Nusselt number, the Prandtl number and the included angle of temperature gradient and velocity vector, respectively.

### 3.4. Reclaimed Carbon Fiber (RCF)-Based Composites

**Figure 12:**
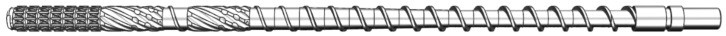
The designed field synergy screw with seven TOEs placed side by side.

We experimentally validated the effect of torsion elements on heat transfer and mixing performance in an extruder during the composite compounding process. More specifically, we studied two screw element configurations: one with screw B fitted with the largest TOEs number and another with screw F where the most dispersed arrangement of TOEs was used. Based on these arrangements, we further optimized the screw design (as shown in Figure 12) to prepare the reclaimed carbon fiber (RCF)-based composites using a single screw extruder. The measured fiber content in composites is 4.5% ± 0.1%.

#### 3.4.1. Fiber Orientation and Distribution

**Figure 13:**
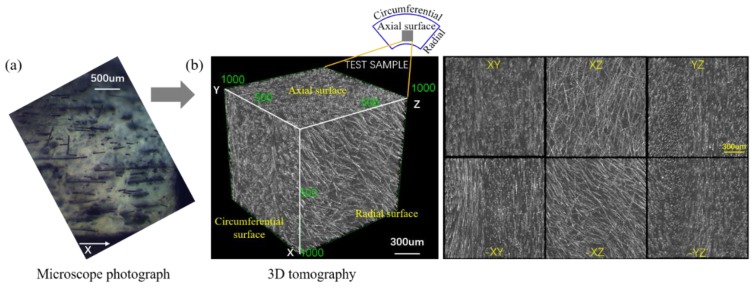
Micromorphology of reclaimed carbon fiber (RCF)/PP: (**a**) Micrograph in cross-section of RCF/PP; (**b**) A 3D representation of the individual fibers in the matrix, Voxel size was (1.48 μm).

Figure 13 shows the cross-section of RCF/PP and a 3D representation of the individual fibers orientations in the matrix (A dynamic 3D display can be found in Video S1). It can be clearly seen that the RCFs are oriented along the flow direction (axial direction), and are evenly distributed parallel to the flow direction (radial and circumferential directions), especially in the radial direction. From the cross-section of the radial surface (XZ and -XZ), we can find that the RCFs were well dispersed in the polymer matrix, which indicates that the torsion element strengthens the radial convection and mass transfer.

**Figure 14:**
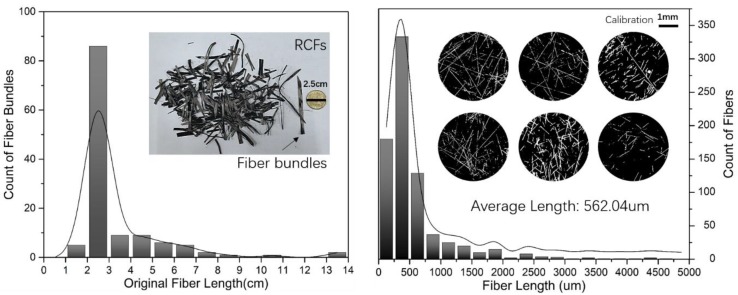
Comparison of fiber length before and after processing.

Figure 14 shows the length distribution of the original fibers and the final fibers. Results showed that most of the original RCFs were about 2–3 cm. After mixing with polymer in the single screw extruder, the average length of RCFs decreased by shear stress in the flow field. However, the average length in the molded specimens was over 500 μm. A number of studies based on glass and carbon fiber composites disclosed a critical fiber length of above 0.3 mm as a requirement to impart the reinforcing effect of fibers in such composites [27,28,29,30]. Therefore, it can be concluded that the field synergy screw used in this study can effectively maintain the length of fibers in the composite matrix while ensuring uniform mixing.

#### 3.4.2. Crystallization Behavior

**Figure 15:**
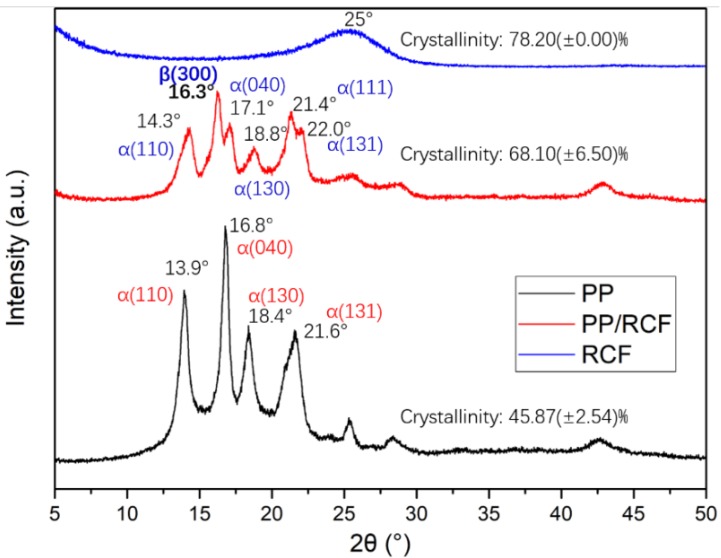
X-ray diffraction (XRD) patterns of PP, RCF and PP/RCF.

Figure 15 shows the X-ray diffraction (XRD) for PP, RCF and PP/RCF composites. It can be found that there are four significant diffraction peaks in the PP diffraction pattern, 2θ equal to 13.9°, 16.8°, 18.4° and 21.6°, which reflects the crystal plane (110), (040), (130) and (131) of *α*-crystalline, respectively [31,32]. Besides these four *α*-crystalline peaks, *β*- crystalline (300) 2θ equals 16.3° and, *α*-crystalline (111) peak 2θ equals 21.4° in the diffraction pattern of PP/RCF composites [31,32]. This indicates that carbon fibers can induce the formation of the *β*-crystalline region in the polypropylene matrix and play a role as a *β*-nucleating agent. We further observed that the crystallinity of PP/RCF composites increases compared with the pure PP material.

#### 3.4.3. Mechanical Properties

**Figure 16:**
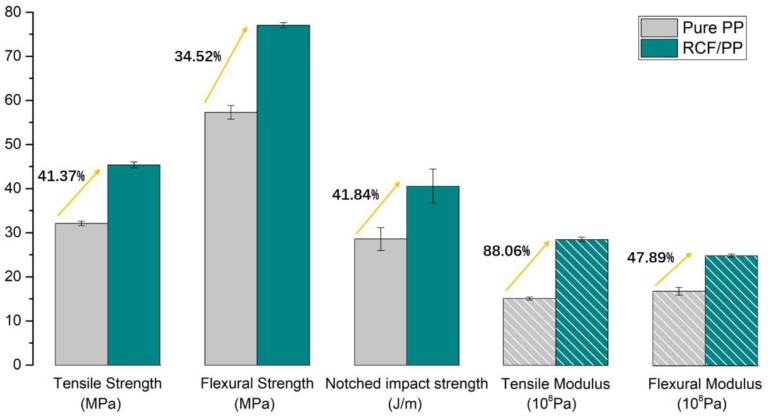
Mechanical properties of PP and PP/RCF.

Analyses of tensile strength, flexural strength and impact strength followed the ASTM D638, ASTM D790 and ASTM D256 standards and the results of mechanical properties are shown in Figure 16. Results indicated that the mechanical properties of PP/RCF composites had been significantly improved compared with that of the pure PP samples, i.e., tensile properties, flexural properties and impact properties were improved by more than 40%, 30% and 40%, respectively. A 40% increase in the impact strength is a significant achievement relative to other conventional reinforcing agents. It is primarily due to that fact that an effective stress transfer under a sudden application of load can be well dissipated to the matrix by carbon fiber due to their high stress transfer capacity, even with a shorter and finer length.

## 4. Conclusions

The effect of bi-phase polymer melt flow with torsional screws compared with a conventional one have been investigated in an extrusion process to examine their heat transfer and mixing performances. Results confirm that the heat transfer and mixing properties of a viscous polymer melt has been enhanced by adapting torsion elements (TOEs) in the screw. Furthermore, the arrangement of TOEs has influence on the heat transfer and mixing mechanisms. The mixing ability and heat transfer properties improve with the increase in the TOE number and the dispersed arrangement of TOEs. Visualization study also revealed that a torsional flow was observed in the TOEs channel, while the flow in the standard elements (STDs) channel was steady without radial movement. This torsional flow resulted in the synergy between the viscous flow field and the thermal flow field, and enhanced the mass and heat transfer to achieve a good thermal management and temperature homogeneity for composites, which is in conformity with the field synergy theory. Besides, the field synergy screw with torsion elements shows good processing behavior, such as good mixing and less-cut of fibers, for preparing the high strength reclaimed carbon fiber-based composites.

## Figures and Tables

**Table 1:** A summary of the physical parameters for polypropylene (PP) and fillers.^1^

Parameter	PP	HIPS	RCFs
Melt flow rate(MFR, ASTM D1238)	2.0–4.0 g/10 min	4.0 g/10 min	-
Vicat softening point(ASTM D1525)	150 °C	95 °C	-
Fiber diameter	-	-	5~8 μm
Fiber length	-	-	2~3 cm

^1^ Data from production companies.

**Table 2:** Geometric parameters of the screw elements.

Dimension in Homogenizing Zone (Hz)	Screw Element	
	STD	TOE
Number of flights	1	12
Width of flights	3	2
Screw diameter (mm)	30	30
Axial screw lead (mm)	30	15
Screw channel height (mm)	1.25	1.75 ^1^

^1^ Maximum value of the TOE channel height.

## Supplementary Materials

The following are available online at https://www.mdpi.com/2073-4360/12/4/771/s1, Video S1: 3D tomography of RCF/PP composites.

## Nomenclature

*t*	= mean residence time: min
*M_s_*	= total weight of extrudates after stopping feeding, kg
*Q_h_*	= flow rate of extruder, kg/min
*D_n_*	= number average particle size, μm
*C_v_*	= coefficient of variation
*σ*	= standard deviation
*μ*	= average value
*Q*	= heat transfer power, W
*A*	= heat transfer area, m^2^
*T*	= melt Temperature, °C
*T_w_*	= wall temperature, °C
*α*	= convective heat transfer coefficient, W/(m^2^·°C)
*Re*	= Reynolds number
*Pr*	= Prandtl number
*Nu*	= Nusselt number
$\bar{U}$	= velocity vector
$\bar{\nabla T}$	= temperature gradient vector
*β*	= field synergy angle
